# Diseases Due to Medical Treatment
*A paper read at the B.M.A. Annual Meeting, Torquay, 21st June 1960.


**Published:** 1961-01

**Authors:** C. Bruce Perry


### DISEASES DUE TO MEDICAL TREATMENT*

BY

C. BRUCE PERRY

The concept that illness may result from medical treatment is not so modern as might at first be thought. Probably cynics have long suspected that this might be so, and in the seventeenth century we find Argan in Molière's play *Le malade imaginaire* saying to Beralde—the serious brother—"Tell me what do you do when you are ill" to which the reply is "Nothing, nothing at all. If you are ill just keep quiet and don't fuss. Let nature take her course, leave her alone to do what is necessary to repair your trouble. It is our own uneasiness and our impatience which is our undoing. Most people die of the remedy not of the disease". However, whatever may have been the state of affairs in seventeenth-century France there has been a great increase in complications of drug therapy during the last thirty years which has coincided with the introduction of new anti-bacterial, anti-convulsant, anti-hypertensive, anti-arthritic, anti-thyroid agents, not to mention tranquillizers and cortico-steroids (Brown, 1959). In fact Moser (1959) has recently published a book on this subject entitled *Diseases of Medical Progress.*

Medical treatment and the general management of the patient may produce illness or disease in various ways and the illness produced may be either organic or psychological. The ill effects may be the result of drugs, physical agents or the doings or sayings of the doctor himself. As this discussion is concerned with illness resulting from medical treatment there is no place for discussing the many and varied unfortunate happenings that may occur to the patient after various surgical procedures except to remind you that they are not unknown. Neither is it proposed to discuss poisoning caused by an accidental or careless overdosage of a drug or the inadvertent administration of the wrong drug. Apart from such "accidents" illness may follow the administration of drugs or therapeutic procedures in various ways.

Toxicity may occur with prolonged administration as for instance with digitalis. Another group of illnesses due to drugs results when the drug is given without sufficient safeguards, in excessive dosage or for too long a time. Crystallisation of acetylated derivatives of sulphonamides in the renal tubules may occur in anyone even if receiving the correct dosage of the drug unless adequate steps are taken to see that the patient does not become dehydrated. The hypo-prothrombinaemia and the resulting haemorrhagic state which may complicate massive salicylate therapy is clearly related to an excessive concentration of the drug in the blood and, of course, forms a considerable hazard to the intravenous administration of salicylates in acute rheumatism, which had such a vogue in certain countries some years ago. More recently fatal cases of nephritis have been described in patients taking phenacetin over a long period of time. One such patient who finally died consumed 3·5 kg. of Phenacetin in five and a half years (Moolten and Smith, 1960). It has been suggested that the renal lesion is due to the long continued methaemoglobinaemia produced by the drug. Such conditions as the pulmonary fibrosis following the prolonged use of hexamethonium for hypertension and the syndrome indistinguishable form systemic lupus erythematosus that occurs so frequently after the continued use of hydrallazines may be similar but are so far unexplained.

The patient may show an abnormal response to a drug which is qualitative in nature —an idiosyncrasy—as when barbiturates give rise to excitement. This is clearly

*A paper read at the B.M.A. Annual Meeting, Torquay, 21st June 1960.

due to an abnormality in the patient, the nature of which is far from clear and which may be related to the differing effect of certain drugs in different species.

All patients vary to some extent in their reaction to drugs and in some this intolerance may be so marked that what would be a small dose for a normal individual produces symptoms of poisoning in the susceptible. It is well known for instance that an occasional patient will develop deafness and tinnitus after a therapeutic dose of quinine. In fact it may be very difficult to decide whether the patient's reaction to the drug is within the range of normal variation or is in fact a manifestation of a real intolerance. Perhaps there is no dividing line. Thus it would appear that the macrocytic anaemia which sometimes complicates the use of anti-convulsants such as phenytoin and even phenobarbitone merely represents one extreme of a constant effect of these drugs. Careful studies of large groups of patients receiving them have shown that while relatively few develop anaemia, in the majority a macrocytosis will be found if this is looked for carefully (Hawkins and Meynall, 1958). However with some drugs severe and devastating results occur in the occasional patient which are not seen in others even after larger doses and long continued administration. For instance some patients can take cinchophen and dinitrophenol with apparent impunity while in others either drug may be rapidly fatal from acute necrosis of the liver. Although in many cases it may be difficult to exclude the possibility of the ill effects resulting from some form of "sensitisation" it is probable that in fact we are dealing with an extreme degree of intolerance. It may be that these unfortunate effects in certain people are due to the fact that patients exhibiting such intolerance have a genetic abnormality resulting in a deficiency of an enzyme which protects non-susceptible individuals. It has been claimed that patients who develop haemolytic anaemia after the administration of primaquine are deficient in a specific enzyme—glucose-6-phosphate dehydrogenase—which normally maintains glutathione in the red cells in a reduced form (Carson *et al*, 1956). This defect has more recently been demonstrated in patients developing haemolytic anaemia after various other drugs including sulphonamides (Szeinberg *et al*, 1957; Beutler, 1959).

However in many cases of the diseases due to drugs there is reason to believe that the underlying mechanism is some form of sensitization (Carr, 1951). It is particularly this type of reaction that is common with the newer synthetic drugs. It is clear that the drug itself, not being a protein, cannot act as an antigen but the drug or one of its degradation products may act as a "hapten" and unite with one of the patient's own proteins which will then be "foreign" and become antigenic. It appears that drugs containing a pyrimidine or "benzamine" grouping are particularly likely to act as a hapten in this way (Samter and Berryman, 1958). There is good evidence for this theory of sensitization in such reactions as are characterized by urticarial rashes, dermatitis, angioneurotic oedema or asthma. Very often there is a history of a previous administration of the drug.

While the basis of this type of sensitivity reaction which follows the second administration of the drug after an interval may be fairly simple to understand, it is more difficult to see exactly what happens in the more common phenomenon where the patient becomes sensitized while still receiving, or still in contact with, the sensitizing agent. Perhaps the best example of this is the long recognized serum sickness, characterized, usually, by urticarial rash, fever, arthritis and adenopathy, occurring about ten days after an injection of foreign serum, and while the causal foreign serum must still be present in the blood stream to excite the reaction. Similar to this are the "drug rashes" and "fevers" seen with so many of our modern drugs, particularly the sulphonamides, and which usually occur five to ten days after the drug was first given even if it has subsequently been stopped. However it is difficult to explain the occasional occurrence of such phenomena after the first dose of a drug in a patient who has never received it previously. It has been suggested that when this happens with penicillin the patient may have become sensitized by exposure to the mould from which penicillin is derived. But it is difficult to explain the fever and massive urticaria which occur not very rarely within a few hours of the first dose of a new drug such as dindevan which the patient can hardly have met previously. Rarely such hypersensitivity reactions may be fatal as when a patient is given a second injection of antitoxin prepared from horse serum some months or years after the first. This serves to remind us that such unhappy events might often be prevented with a little care and forethought. For instance when analysing thirty fatal reactions to penicillin Rosenthal (1958) found that in fourteen no enquiry had been made as to whether the patient had ever received penicillin before, and in ten the patients had in fact had lesser reactions to penicillin on previous occasions.

While discussing this problem of sensitization to drugs it is perhaps worth while stressing the fact that sensitization is far more likely to occur when the drug is given by some routes than by others. Thus penicillin and sulphonamide sensitization occur relatively frequently if the drug is applied to the skin, and fortunately comparatively infrequently when administered by mouth or by injection. For this reason it is always preferable to treat skin sepsis by systemic rather than local administration of these drugs. Similar sensitization may follow the surface application of local anaesthetics such as benzocaine, yet the subcutaneous administration of the same substance seems relatively immune from this risk.

However, many of the diseases which follow administration of drugs and which we believe to be due to sensitization are less easy to understand and are far more complex. There seems to be little doubt that polyarteritis nodosa has followed second administration, after an interval, of both sulphonamides and thiouracils. While we can understand how the drug assumes antigenic properties by becoming conjugated with one of the patient's own proteins it is difficult to see why the arteries in this particular case are singled out. Ackroyd's work (1953) on thrombocytopenic pupura following sedormid has shown clearly that sedormid causes platelet lysis in the blood of sensitized patients and it would appear that sedormid unites with platelets to constitute an antigen which stimulates the formation of antibody which in the presence of complement lyses the antigen (i.e. the "sedormized" platelets). It is probable that this combination always happens whenever sedormid comes into contact with platelets. The fact that only a few of the patients who take the drug develop purpura may be due to the compound being a substance of low antigenicity which stimulates antibody formation only in patients whose immunity reactions are readily stimulated by so weak an antigen. The work of Bolton (1956) demonstrates that a similar mechanism operates in thrombocytopenia due to quinine, and there is good reason to suppose that the thrombocytopenia that follows the administration of some other drugs has the same basis. The experiments of Steinkamp, Moore and Doubek (1955) show that the antibody in a patient with thrombocytopenic purpura due to quinine can be transferred passively in the patient's serum so that a recipient who is taking quinine develops thronbocytopenia and purpura. The nature of the jaundice which may follow the use of chlorpromazine and similar "tranquillizers" and which was originally thought to be due to a fairly benign cholangiolytic process but which recent work shows is in fact associated with necrosis of liver cells (Zelmann, 1959) is not altogether clear. But the following case history strongly suggests that it too is a sensitization phenomenon. A patient with recurrent attacks of endogenous depression was referred some two years ago for unexplained recurrent jaundice. She first developed jaundice after receiving chlorpromazine for some time. The drug was stopped and the jaundice cleared but the association between the two was not recognized. Some six months later there was a return of depression and chlorpromazine was again prescribed but this time jaundice developed after the second dose of the drug.

Another group of illnesses due to treatment is that comprising diseases which result from side effects of drugs. We have long recognized the dry mucous membranes that follow the use of atropine in full doses, and the somnolence caused by antihistamines is so well recognized that they are now sometimes used as mild hypnotics. Antibiotics by destroying useful commensals in the alimentary canal in addition to the pathogenic organisms giving rise to the disease for which they are prescribed may give rise to the troublesome ano-rectal syndrome, may allow monilia to proliferate or, even more serious, allow resistant staphylococci to flourish and so produce a fatal enteritis. The emergence of drug resistant organisms and the occurrence of illness due to infection with the organisms is clearly another example of undesirable side effects of the use of invaluable antibiotics. Similar undesirable "side effects" are probably responsible for the severe depression that may follow the use of rauwolfia and its derivatives. Chlorpromazine and its allies may in the same way give rise to Parkinsonism, which may so closely mimic that due to encephalitis lethargica as to be associated with oculo-gyral crises. In the same way the therapeutic use of steroids may produce a clinical picture closely resembling Cushing's syndrome, perforation of peptic ulcer, osteoporosis with collapse of vertebrae and the masking of intercurrent infection. Another troublesome complication of steroid therapy is the exacerbation of the treated disease that so commonly follows withdrawal of the drug. But this phenomenon is not peculiar to the steriods for it may also occur, but usually to a lesser degree, when salicylates are stopped in patients with acute rheumatism. A much more serious effect of prolonged steroid therapy is the severe depression of suprarenal function which makes the patient extremely vulnerable to any stress in the shape of infection, anaesthesia or surgical operation (Allanby, 1957; Bayliss, 1958; Dundee, 1958). Another example of the results of successful "treatment" giving rise to new illness is the haemorrhagic state that may occur with anticoagulant therapy, which may cause for example haemorrhagic pericarditis in patients with mycordial infarction, or bleeding from an unsuspected peptic ulcer. Similarly the successful reduction of blood pressure by hypotensive drugs may precipitate the onset of renal failure (Carter and Payne, 1956) or myocardial ischaemia (Judson *et al*, 1956) in patients whose renal or coronary circulation has only been adequately maintained hitherto by means of the hypertension.

The use of X-rays and other radio-active agents may also cause disease. Whether or not X-ray therapy affects the course of ankylosing spondylitis there seems to be little doubt that patients so treated are far more likely to develop leukaemia than the normal population. Twenty years ago there was a vogue for the irradiation of babies with supposedly enlarged thymus, especially in the United States, and this has been followed by a slight but definite increase in the incidence of carcinomata and adenomata of the thyroid and of leukaemia in these patients. (Simpson *et al.*, 1955). Similar to this is the malignant disease which so frequently followed the use of thorotrast as a contrast medium in radiology. Fortunately this hazard is now so well known that its use for this purpose has been discarded.

A further group of diseases due to treatment arises from the excessive use of substances which in moderation may be beneficial and which have previously been considered harmless, on the view that one cannot have too much of a good thing. A clear example of this is the tragedy of retrolental fibroplasia with resulting blindness which we now realise was due to exposing premature babies to too high a concentration of oxygen. The assumption that vitamins are always beneficial, no matter in what dose they may be taken, has probably been responsible for more than one "new" disease. There is good reason to believe that the severe jaundice giving rise to kernicterus in premature babies is largely due to the over enthusiastic use of "synkavit" given in an attempt to avoid the possible risk of haemorrhagic disease of the newborn. So too nephrocalcinosis, another new disease of infancy, may well be the result of poisoning with vitamin D which, as well as being enthusiastically prescribed by doctors, was at one time added to all sorts of infant foods by the manufacturers. One other example of disease resulting from the administration of apparently harmless drugs is pink disease which was once so common in paediatric practice and the nature and cause of which was so baffling. The recognition that this might be the result of chronic poisoning with mercury often given in the shape of proprietary "teething powders" led to the mercury being omitted from such powders and the sudden disappearance of the disease.

So much for a few of the diseases that may follow the administration of drugs; but before concluding I would like to refer to two other ways in which treatment may give rise to disease. Firstly it is important to remember that infection may be spread by the doctor in various ways. A very important example is that of homologous serum jaundice which as far as I am aware is only known to occur as a sequel to intravenous injection or withdrawal of blood. It was a long time before we realized that the jaundice which we so happily attributed to the "toxic" effect of novarsenobenzol was not due to the drug, but to the fact that the syringe or needle had not been properly sterilized and that with the injection of the drug the patient also received a minute dose of blood left in the syringe from a previously injected patient. It is not so very long ago that Semmelweiss showed how puerperal fever was carried by the obstetrician's hands. No doubt we are all now well aware of such dramatic possibilities, but I sometimes wonder whether we may not still be helping to spread infection. For instance Ridley (1958) has described the contamination of eye-drop bottles with bacteria and Phillips (1960) mentions epidemics of kerato-conjunctivitis spread by drop bottles.

Finally I would draw your attention to psychological illnesses that may be caused by the way the doctor either handles or talks to his patient. During the war it was not uncommon to see young men who had developed the symptoms of the effort syndrome a few weeks after being rejected for military service without their being told the reason. This was especially so if during the examination they thought that any special attention had been paid to auscultation of the heart. Many an anxiety state is induced too by the facile statements made to patients by well meaning doctors to the effect that they have a "touch of high blood pressure". The patient ignores the significance of the "touch" which is meant to be reassuring but the fear of the high blood pressure fairly soon gives rise to headache, giddiness and palpitations, so often considered by patients and also sometimes by doctors as the typical manifestation of high blood pressure. Whether or not there is such a "disease" as high blood pressure, there is little doubt that very many patients suffer not from their abnormal cardiovascular responses but from being told by a doctor that their blood pressure is high. So too tragedy may follow the faulty handling of patients with coronary artery disease or its incorrect diagnosis. Many years of needless invalidism or even worse may be caused in this way. A good example is the patient described by William Evans (1959). A man aged 47 was diagnosed as having a myocardial infarction and treated by six weeks rest in bed and further convalescence. At the end of this time his employers refused to take him back as a bus driver and try as he would he could not obtain work. He became more and more depressed and finally committed suicide by drowning. The necropsy performed for the coroner revealed widely patent coronary arteries and a healthy myocardium. We should therefore always remember when talking to patients the possibility of inducing an anxiety state by an incautious or injudicious statement and be as careful over what we tell a patient about his symptoms as we should be in prescribing potentially harmful drugs for possibly trivial symptoms.

### REFERENCES

Ackroyd, J. F. (1953). *Amer. J. Med.*, **14**, 605.
Allanby, K. D. (1957). *Lancet*, *i* 1104.
Bayliss, R. I. S. (1958). *B.M.J.*, *ii*, 935
Beutler, E. (1959). *Blood*, **14**, 103.
Brown, E. A. (1958). Proceedings of the IIIrd International Congress of Allergy. Paris 1958 p.269.
Bolton, F. G. (1956) *Blood*, **11**, 547.
Carr, E. A. (1951) *New Eng. J. Med.*, **245**, 892.
Carson, P.E., Larkin, F., Ickes, C. E. and Alving, A. S. (1956) *Science*, **124**, 484.
Carter, A. B. and Payne, R. W. (1956). *Lancet*, *ii*, 537.
Dundee, J. W. (1958). *B.M.J.*, *i*, 1434.
Evans, W. (1959). *B.M.J.*, *i*, 249.
Hawkins, C. F. and Meynell, M. J. (1958). *Q.J.M.*, **27**, 45.
Judson, W. E., Hollander, W. and Wilkins, R. W. (1956). *Circulation*, **13**, 533.
Moolten, S. E. and Smith, I. B. (1960). *Amer. J. Med.*, **28**, 127.
Moser, R. M. (1959). *Diseases of Medical Progress*, Springfield, III.,
Phillips, C. I. (1960). Personal communication.
Ridley, F. (1958). *Brit. J. Opthalmology*, **42**, 641.
Rosenthal, A. (1958). *J. Amer. Med. Assoc.*, **167**, 118.
Samter, M. and Berryman, G. H. (1958). Proceedings of the IIIrd International Congress of Allergy, Paris 1958, p.261.
Simpson, C. L., Hempelmann, L. H. and Fuller, L. M. (1955). *Radiology*, **64**, 840.
Steinkamp, R., Moore, C. V. and Doubek, W. G. (1955). *J. Lab. & Clin. Med.*, **45**, 18.
Szeinberg, A., Sheba, C., Hirshorn, N. and Bodonyi, E. (1957). *Blood*, **12**, 603.
Zelman, S. (1959). *Amer. J. Med.*, **27**, 708.

